# Pilot study: use of gallium-68 PSMA PET for detection of metastatic lesions in patients with renal tumour

**DOI:** 10.1186/s13550-016-0231-6

**Published:** 2016-10-22

**Authors:** Handoo Rhee, John Blazak, Chui Ming Tham, Keng Lim Ng, Benjamin Shepherd, Malcolm Lawson, John Preston, Ian Vela, Paul Thomas, Simon Wood

**Affiliations:** 1 Department of Urology, Princess Alexandra Hospital, Brisbane, QLD 4102 Australia; 2 Pathology Queensland, Princess Alexandra Hospital, Brisbane, Australia; 3 Department of Nuclear Medicine, Royal Brisbane and Women’s Hospital, Brisbane, Australia; 4 University of Queensland, School of Medicine, Brisbane, Australia

**Keywords:** PSMA, PET, Renal cell carcinoma, Metastasis, Nephrectomy

## Abstract

**Background:**

In this study, we prospectively evaluate the diagnostic potential of a gallium-68 (68Ga) prostate-specific membrane antigen (PSMA)-binding ligand and positron emission tomography (PET) in detecting metastatic lesions in patients with renal tumour. The secondary aim was to determine whether the findings would result in the alteration of patient management.

**Results:**

Ten patients with renal lesion and potential metastatic disease on conventional imaging were recruited. Patients underwent PSMA PET in addition to standard imaging. Nine patients underwent nephrectomy and 4 patients underwent additional targeted biopsy to provide specimens for histopathological validation. There were 89 pathological lesions on CT, of which 32 were removed or biopsied for histopathological correlation. With PSMA PET, 86 PET avid lesions were identified with 36 samples being available for analysis. Thirty-five of 36 samples were positive for renal cell carcinoma deposits, whilst 1 sample was inconclusive for diagnosis on biopsy. For the histologically confirmed lesions, there were no false-negative PSMA PET lesions; however, CT was false negative in 11. In two patients, surgical strategies were changed based on PSMA PET findings.

**Conclusions:**

PSMA PET may potentially have a role in the preoperative staging of advanced renal cell carcinoma as PET detected multiple histologically proven metastatic lesions which were false negative on CT scanning, resulting in change in surgical strategies in some patients. We cautiously support a larger study to confirm these results and to assess the longitudinal impact on patient outcomes.

**Trial registration:**

Australia and New Zealand Clinical Trial Registry (ANZCTR), ACTRN12615000854538.

**Electronic supplementary material:**

The online version of this article (doi:10.1186/s13550-016-0231-6) contains supplementary material, which is available to authorized users.

## Background

Kidney cancer is the sixth most common cancer in men and the 11th most common cancer in women in Australia. In 2014, metastatic renal cell carcinoma (RCC) accounted for approximately 2 % of cancer death [1]. Patients who are diagnosed with localized disease are usually offered radical or partial nephrectomy. Unfortunately, depending on the tumour characteristics such as histologic type, nucleolar grade, stage and adverse pathological features, up to 50 % of patients with clinically localized disease will develop metastasis during follow-up [2, 3]. In patients later diagnosed with metastatic disease, prognosis is poor with 5-year survival rate below 10–20 % [4, 5].

During the initial staging, TNM classification by the American Joint Committee on Cancer (AJCC) is used to define metastatic disease [3]. Using these criteria, in early phases of metastatic disease, neither computed tomography (CT) nor bone scan (BS) is sensitive in detecting small metastatic lesions [6]. There is a significant clinical need for the development of more sensitive and specific imaging technology to detect metastatic foci that could lead to early treatment and potential cure in true oligometastatic settings. Magnetic resonance imaging has been used to help further characterize renal tumours, tumour extension, locoregional lymph nodes and metastatic disease, although it is not used routinely [7].

Positron emission tomography (PET) is a technology that may improve metastasis detection and challenge the size criteria used for determining metastatic nodal involvement by conventional imaging. PET can be used to locate lesions with particular metabolic parameters or expression of specific surface markers. For example, fluorodeoxyglucose (FDG) has been used for staging, prognostication and follow-up [8, 9]. It has previously been demonstrated that a type II integral membrane glycoprotein highly expressed in prostate cancer cells called ‘prostate-specific membrane antigen’ (PSMA) is also up-regulated in the neovasculature of solid tumours including RCC [10]. For clear cell RCC, the reported PSMA expression ranges from 80 to 100 %, whilst in other carcinoma types such as chromophobe and papillary, the expression is not as common (30–60 and 0 %, respectively) [10–13]*.* Recently, Rowe et al. and Gorin et al*.* demonstrated promising PET results with a novel PSMA-binding ligand, 18F-DCFPyL, for detection of metastatic renal cell carcinoma [14, 15]. A recent case report also demonstrated significant improvement in staging metastatic clear cell RCC using another novel PSMA-binding ligand gallium-68 (68Ga)-PSMA-HBED-CC, over FDG PET or CT imaging [16].

In this study, we prospectively evaluate the diagnostic potential of PET using 68Ga-PSMA-HBED-CC (PSMA PET) in detecting metastatic lesions in patients with renal tumours with the secondary aim of determining whether the findings will result in the alteration of treatment decisions.

## Methods

### Study design and population

Following ethical clearance, a phase I pilot clinical trial was conducted (*Ethics Approval Number: HREC/15/QPAH/292*, http://www.anzctr.org.au/default.aspx *: ACTRN12615000854538*). All patients provided informed consent prior to enrolment. Ten consecutive patients (*n* = 10) newly diagnosed with renal tumour and suspicion for metastatic disease on standard imaging were recruited into the trial. Patients were considered to harbour metastatic lesions according to RECIST criteria 1.1: Measurable lesions defined as lymph nodes greater than or equal to 10 mm in short axis, or tumour lesions with minimum size of 10 mm by CT scan, or 20 mm by chest X-ray [17]. Those who were unable to lie flat and had prior history of other malignancies within the last 2 years, end-stage renal failure or on haemodialysis were excluded from the study.

### Standard imaging

In all patients, the primary renal tumour was identified on computed tomography. Magnetic resonance imaging (MRI), ultrasound (US) and bone scan (BS) were available in some cases for correlation. CT images were performed on either Siemens Somatom Definition Flash (2 × 192 slices) or Philips Brilliance iCT (256 slices). An experienced uro-radiologist reported the imaging findings prior to surgery and was blinded to the results of the PSMA PET images. Patients were reported and staged according to TNM staging and RECIST 1.1 criteria [17]. Two patients were unable to receive iodine contrast due to renal impairment, and one was allergic to gadolinium for MRI.

### PSMA PET

PSMA PET was performed within 4 weeks (median delay = 3 weeks, range 1–4 weeks) of obtaining standard imaging. 68Ga-PSMA-HBED-CC (HBED-CC, ABX AG, Germany), also known as 68Ga-PSMA-11, was manufactured at the Specialised PET Services Queensland Radiopharmaceutical laboratory as per Eder et al*.* [18]. PET images were acquired 60 min after administration of 150 MBq ± 5 % of 68Ga-PSMA-HBED-CC for 3 min per bed position on a Siemens Biograph mCT FLOW PET/CT scanner. Iterative PET image reconstruction was performed using 21 subsets, 3 iterations and matrix size of 200. A low-dose computed tomography (CT) scan was performed with the PET scan for anatomic localisation and attenuation correction. Combined PET/CT images were read by an experienced nuclear medicine physician. Lesions of interest were considered positive by qualitative visual assessment, where avidity was greater than background in areas without physiological uptake. For example, a small lymph node with PET avidity greater than 1.5 times greater than background was recorded as pathological regardless of its size.

### Histopathologic analysis

Ex-vivo histopathologic analysis was independently performed by a single experienced uropathologist. The resected samples were formalin-fixed and paraffin-embedded into tissue blocks. Tissue slides were cut from the blocks and stained with haematoxylin and eosin for histopathologic evaluation.

### Surgery

Of ten patients, nine patients underwent radical nephrectomy with removal of regional lymph nodes and putative malignant lesions. One patient was found to be not suitable for surgery due to obstructed superior vena cava from large mediastinal nodes. Operations were performed by three experienced urological surgeons who were guided by conventional imaging and PSMA PET.

### Statistical analysis

The radiologist, the nuclear medicine physician and the uropathologist were blinded to the results of the individual components of the study. Histopathology reports were used as reference to perform statistical calculations where possible. The reports composed of dimensions, location and characteristics of renal and extra-renal lesions. Sensitivity, specificity, positive predictive value and negative predictive value were calculated using SPSS (IBM Corp. Released 2013. IBM SPSS Statistics for Windows, Version 22.0. Armonk), and presented as 95 % confidence intervals (CI).

## Results

### Patient characteristics

Between August 2015 and January 2016, ten consecutive patients with metastatic lesions and renal tumour were enrolled into the study (Table 1). All patients underwent standard imaging such as CT with or without MRI/US/BS (Additional file 1: Table S1). All ten patients were males, with the median age of 57 ± 12.2 years. Most patients had a large primary tumour with the median size of 7.8 ± 4.3 cm.

**Table 1:** Patient characteristics

Characteristics	Parameters		
Male/female	10:0		
Age—median	57 ± 12.2 years		
Primary tumour size—median	78.3 ± 42.6 mm		
Regional lymph nodes on CT/MRI	*n* = 3 (30 %)		
Pulmonary lesions on CT/chest X-ray	*n* = 6 (60 %)		
Bone lesions on CT/MRI/BS	*n* = 3 (30 %)		
Staging—pathological TNM (post-operative or biopsy)	Primary tumour T2: *n* = 2 T3: *n* = 5 T4: *n* = 3	Lymph node N0: *n* = 8 N1: *n* = 2	Metastasis M0: *n* = 5 M1: *n* = 5
Final histopathology	8 clear cell RCC 1 papillary 1 unclassified		

*Abbreviations*: *BS* bone scan, *CT* computed tomography, *M* metastasis, *MRI* magnetic resonance imaging, *n* number, *N* lymph node, *RCC* renal cell carcinoma, *T* tumour

### Computed tomography

Using CT of the chest and abdomen, 89 lesions were identified overall (78 extra-renal lesions). Thirty-two CT-identified lesions were surgically removed or biopsied for histopathological correlation. Of the lesions, 24 were consistent with renal cell carcinoma (Additional file 1: Table S1). The diagnostic values calculated from histological samples were as follows: sensitivity 68.6 % (95 % CI 51–83 %) and positive predictive value (PPV) 80 % (95 % CI 61–92 %). When resected samples were considered as true negatives (e.g. non-pathological adrenal gland, regional lymph nodes or biopsy sample), specificity and negative predictive value (NPV) were 88.46 % (95 % CI 76–95 %) and 80.70 % (95 % CI 68–90 %), respectively (Table 2). The findings were consistent with the current literature [19]. Two of the ten patients did not receive contrast due to severe renal impairment from large renal tumours and obstructing IVC thrombi, limiting the efficacy of CT.

**Table 2:** Diagnostic values of CT and PSMA PET

	CT	PSMA PET
Lesions detected	89	86
Sensitivity	68.6 % (CI 0.51–0.83)	92.11 % (CI 0.78–0.98)
Positive predictive value	80 % (CI 0.61–0.92)	97.22 % (CI 0.84–1.00)
Positive likelihood ratio	3 (1.59–5.65)	35 (5.06–241.94)
TNM staging	T2 = 2 T3 = 8 T4 = 0 N0 = 8 N1 = 2 M0 = 3 M1 = 7	T2 = 2 T3 = 7 T4 = 1 N0 = 6 N1 = 4 M0 = 2 M1 = 8

*Abbreviations*: *CT* computed tomography, *M* metastasis, *n* number, *N* lymph node, *PSMA* prostate-specific membrane antigen, *RCC* renal cell carcinoma, *T* tumour

### PSMA PET

There were 86 PSMA PET abnormalities reported as primary or metastatic lesions. Histological correlation was available for 36 of these lesions with 35 of these demonstrating renal cell carcinoma deposits, whilst 1 biopsy of pancreatic tail lesion was found to be inconclusive. For the histologically confirmed lesions, there were no false-negative PSMA PET lesions; however, CT was false negative in 11. In the primary lesion, average maximum standardized uptake value (SUVmax) was 18.0 (range 3.7–36.5), whilst the average SUVmax for metastatic foci was 19.5 (range 1.5 to ±48). For patient 5 with papillary RCC, primary and renal vein thrombus SUVmax were lower than in others at 3.6 and 5.1, respectively. In patient 3 with ccRCC and sarcomatoid differentiation, primary tumour SUVmax was 28.6. In patient 7 with unclassified RCC, primary tumour SUVmax was 18.3.

### PSMA PET led to alteration in patients’ management

**Fig. 1:**
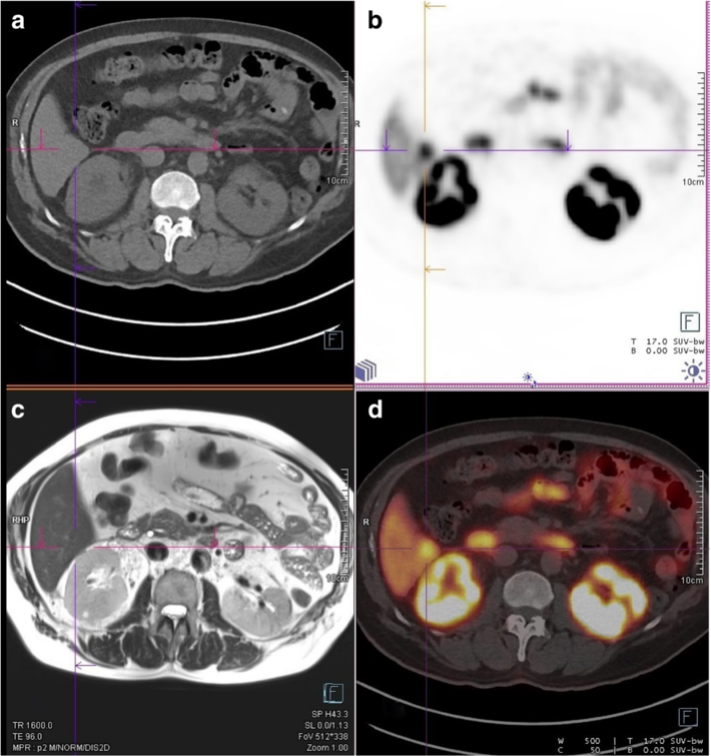
Comparison of non-contrast CT, MRI and PSMA PET in subject 2. **a** Non-contrast CT or **c** non-contrast T1 sequence of MRI did not reveal a lesion in the right lobe of the liver. On **b** and **d** which are PSMA PET images, a focal liver lesion was identified with SUVmax of 15.3 and dimensions of 17 × 13 × 14 mm. **d** Fused PET and CT images. The patient had moderate renal impairment and contrast allergy, prohibiting intravenous contrast with MRI or CT

**Fig. 2:**
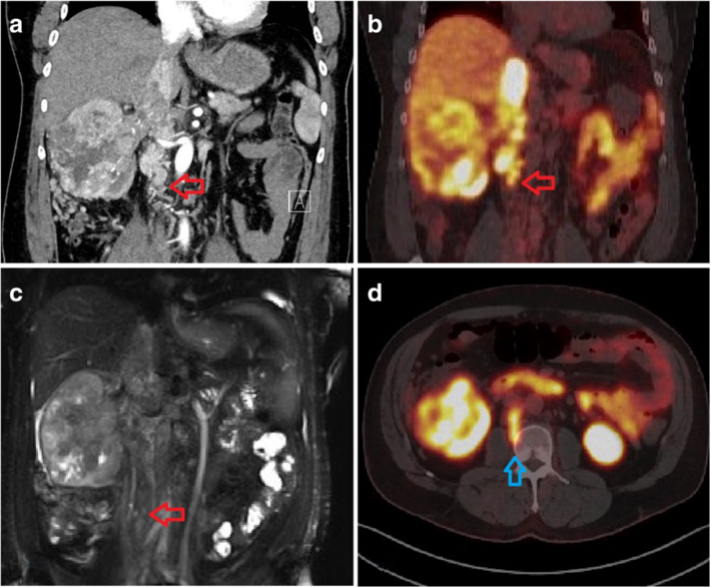
Comparison of CT, MRI and PSMA PET in subject 1. **a** Contrast CT demonstrates large renal lesion with IVC thrombus extending down to bilateral lower limbs. **b** PSMA PET demonstrates avidity within primary tumour and tumour thrombus down to the level of bland thrombus (*red arrow*). **c** MRI showing tumour within IVC, surrounded by bland thrombus. **d** Axial PSMA PET demonstrates tumour thrombus extending into the lumbar vein, which was not identified by other imaging modalities—*blue arrow*

In one patient (Fig. 1—subject 2), a small liver metastatic lesion was identified with PSMA PET that was not revealed on non-contrast MRI, US or CT. The patient was recorded to have previous contrast and gadolinium reaction, and had non-contrast MRI of the abdomen and liver, which limited the benefits of CT or MRI. The patient underwent subsequent cytoreductive nephrectomy and hemihepatectomy (with histological confirmation of the liver metastasis). In subject 1 (Fig. 2), bland tumour was demonstrated in inferior vena cava below tumour thrombus that was highly PET avid. At the same time, there was an extension of tumour thrombus into lumber vein that was identified on PET only. The bland thrombus was transected below viable tumour, and the viable tumour with lumbar vein thrombus was excised using PET imaging as the guide (with histological confirmation of tumour involvement corresponding to the PET abnormality).

## Discussion

In this pilot study, we have compared the diagnostic value of PSMA PET with conventional imaging such as CT in patients with metastatic RCC. In comparing the results of imaging to the histopathological reference from surgical excisional or biopsy samples, PSMA PET appears to provide comparable sensitivity and PPV over standard imaging modalities. It resulted in two patients having treatment modified based on the results. Moreover, it has the advantage of being able to be used in patients with renal impairment or contrast allergy where the administration may be contraindicated.

The greatest advantage of PSMA PET over standard CT is its ability to identify small lesions or lesions in areas where visualization is difficult such as in the liver especially when contrast cannot be used. According to the RECIST criteria 1.1, the dimensions of a malignant lymph node is defined as a node greater than 15 mm in short axis depending on the parts of the abdomen and pelvis [17]. Using PSMA PET, the smallest node identified was 6 mm with SUVmax of 3.1. Similarly, within the lung fields, we identified 34 lung lesions with the average short axis of 9.6 mm and SUVmax of 4.6. Unfortunately, there was only 1 histopathological sample from the lung fields available for correlation. Being able to identify sub-centimetre lesions may be important for patients with true oligometastatic disease or those with planned cytoreductive nephrectomy where the adjacent tumour deposits may be removed at the same time. In addition, lesions found outside surgical fields may be targeted using stereotactic ablative radiotherapy.

The evidence for using PSMA expression in the neovasculature of renal cell carcinoma deposits for imaging has been building recently. In a recent case series by Sawicki et al., the authors demonstrated in six patients the ability of 68Ga PSMA ligand and PET to detect metastatic lesion with high contrast; however, overall SUVmax was substantially lower in primary disease (0.2 ± 0.3) [20]. Using another PSMA-targeting ligand 18F-DCFPyL, Rowe et al*.* demonstrated superiority of the ligand over conventional imaging with similar sensitivity (94.7 %) in five patients [14]. Further, Gorin and Rowe et al. followed up the findings with the results from a rapid autopsy where seven of eight sites of radiotracer uptake that was not demonstrated on contrast enhanced CT were biopsied and confirmed to be positive for ccRCC.

PSMA is a functional enzyme that may have a role in developing neovasculature in solid tumours. Rowe et al*.*, therefore, also considered the utility of SUVmax calculations in lesions as a prognostic indicator of response to systemic therapy such as tyrosine kinase inhibitors (TKI). For example, metastatic clear cell RCC patients are more likely to respond to TKI than with another subtype of RCC that are less likely to express PSMA. Using the same ligand, Gorin et al*.* performed a rapid autopsy after administration and found that all 98 % of CT-characterized lesions from a patient with metastatic disease were visualized on PET/CT with 12 further lesions being found. All histologically proven sites of ccRCC demonstrated PSMA expression [15]. Another ligand of interest is indium-111-labelled J591 anti-PSMA antibody. Pandit-Taskar et al*.* recently published the results of 5 case series of patients with metastatic RCC and 15 with other types of solid tumours in phase I clinical trial [21]. In the study, nodal lesion detection rate was 66 % in patients with metastatic RCC.

The current study aims to determine the clinical benefits of PSMA PET over standard imaging in patients with metastatic renal cell carcinoma. The study was limited in that not all the suspected metastatic lesions on CT and PET underwent histologic confirmation. Removing PET avid lesions selectively during surgery has introduced selection bias to the calculation of diagnostic values, and it is one of the major flaws of the study. Further, no reference standard for PET or CT negative lesions was used. Nevertheless, over one third of the suspected lesions had histologic confirmation and 11 out of 36 of these were false negative on CT imaging (with no false negatives on PET). In two patients, contrast CT was unable to be used due to renal impairment, potentially favouring the outcomes of PSMA PET in these cases.

It is also worth noting that PSMA expression is not specific to prostate or ccRCC. Therefore, caution must be exercised in interpreting the results in those with dual pathologies. Further, due to urinary excretion of 68Ga and PSMA expression in proximal tubules of kidney, there is limitation in using PSMA PET for detection and characterization of primary renal tumours.

## Conclusions

This pilot study has demonstrated that PSMA PET may potentially have a role in the preoperative staging of advanced renal cell carcinoma as PET detected multiple histologically proven metastatic lesions which were false negative on CT scanning. Surgical strategy was changed in two patients based on PSMA PET results, with the PET results subsequently confirmed as true positive. We cautiously support a larger study to confirm these results and to assess the longitudinal impact on patient outcomes.

## Additional file

Additional file 1: Table S1. Results of radiological imaging. (DOC 41 kb)
