# Supplementary material for: Pilot study: use of gallium-68 PSMA PET for detection of metastatic lesions in patients with renal tumour
Source: EJNMMI Res. 2016 Oct 22;6:76. doi: 10.1186/s13550-016-0231-6 (PMC5075321; doi:10.1186/s13550-016-0231-6)
Supplement: Additional file 1: Table S1. — Results of radiological imaging. (DOC 41 kb) [file 13550_2016_231_MOESM1_ESM.doc]

**Additional file 1: Table S1**. Results of Radiological Imaging

| **Subject Number** | **CT lesions** | **Histopathology positive lesions on CT** | **PSMA lesions** | **Histopathology positive lesions on PSMA PET** | **Final Histopathology** |
| --- | --- | --- | --- | --- | --- |
| 1 | N=14  5 retrocaval nodes  5 pulmonary lesions  1 kidney lesion  1 renal vein lesion  1 IVC lesion  1 Adrenal gland | 9 out of 9 (kidney, lymph nodes, adrenal, renal vein, IVC)  Pulmonary lesions not assessed | N=20  5 retrocaval nodes  5 aortocaval nodes  5 pulmonary lesions  1 kidney lesion  1 renal vein thrombus  1 IVC lesion  1 lumbar vein thrombus  1 Adrenal lesion | 15 out of 15 (kidney, lymph nodes, adrenal, renal vein, lumbar vein, IVC)  Pulmonary lesions not assessed | Clear cell RCC pT4, nucleolar Grade 4, 10 out of 10 lymph node positive. Tumour thrombus in renal vein, IVC. Tumour deposit in adrenal gland |
| 2 | N=3  2 retroperitoneal nodes  1 kidney lesion  Non-contrast | 1 out of 3  (kidney lesion) | N=5  1 Liver lesion  1 kidney lesion  1 renal vein thrombus  1 IVC thrombus  1 adrenal lesion | 5 out of 5 (kidney, IVC, liver, adrenal, renal vein) | Clear cell RCC, pT4, nucleolar grade 3, 0 out of 10 lymph nodes positive. Perineural invasion and microvascular invasion. Metastasis to liver. Tumour thrombus in renal vein, and IVC. |
| 3 | N=16  15 pulmonary lesions  1 kidney lesion | 2 out of 2  (lung and kidney)  Not all pulmonary lesions assessed | N=16  15 pulmonary lesions  1 kidney | 2 out of 2 (lung, kidney)  Not all pulmonary lesions assessed | Clear cell RCC, pT2a, nucleolar grade 4, sarcomatoid differentiation  Lung biopsy positive for clear cell RCC. |
| 4 | N=2  2 kidney lesions | 2 out of 2 (kidney) | N=3  2 kidney lesions  1 pancreatic lesion | 2 out of 3 (2 kidney lesions) | Two renal lesions: both clear cell RCC, nucleolar grade 2  Pancreatic tail lesion FNA via EUS |
| 5 | N=2  1 kidney lesion  1 adrenal lesion  Non-Contrast | 2 out of 2 (kidney and adrenal) | N=7  3 bone lesions  1 kidney lesion  1 renal vein thrombus  1 adrenal lesion  1 pulmonary artery lesion | 3 out of 3 (adrenal, kidney, renal vein)  Bone and pulmonary artery lesions not assessed | Papillary RCC, pT4, nucleolar grade 4, lymph nodes 2 out of 6 positive. Lymphovascular invasion. Metastasis to adrenal gland. |
| 6 | N=6  2 retroperitoneal nodes  1 kidney lesion, 1 adrenal lesion, 2 lung lesions | 1 out of 6 (kidney) | N=1  1 kidney lesion | 1 out of 1 (Kidney) | Clear cell RCC, pT3a, nucleolar grade 4, lymph nodes 0 out of 22 positive. |
| 7 | N=24  1 bone lesion  20 pulmonary lesions  1 kidney lesion  1 renal vein thrombus  1 adrenal lesion | 2 out of 2  (kidney and renal vein)  Pulmonary lesions not assessed | N=6  4 pulmonary lesions  1 kidney lesion  1 renal vein thrombus | 2 out of 2 (kidney and renal vein)  Pulmonary lesions not assessed | Clear cell RCC, pT3a, nucleolar grade 4, lymph nodes 0 out of 12 positive. |
| 8 | N=16  3 bone lesions  11 pulmonary lesions  1 kidney lesion  1 adrenal lesion | 1 out of 1 (manubrium)  Kidney and other metastatic lesions not assessed | N=17  5 bone lesions  5 retroperitoneal lesions  5 pulmonary lesions  1 kidney lesion  1 adrenal lesion | 1 out of 1 (manubrium)  Kidney and other metastatic lesions not assessed | Bone biopsy – unclassified RCC. |
| 9 | N=2  1 kidney lesion  1 renal vein thrombus | 2 out of 2 (kidney, renal vein) | N=2  1 kidney lesion  1 renal vein thrombus | 2 out of 2 (kidney, renal vein) | Clear cell RCC, pT3a, nucleolar grade 2, lymph nodes 0 out of 2 positive. |
| 10 | N=4  1 bone lesion  2 pulmonary lesions  1 kidney lesion | 2 out of 2 (T4 spine and kidney)  Pulmonary lesions not assessed | N=9  1 bone lesion  1 retroperitoneal lesion  5 pulmonary lesions  1 kidney lesion  1 quadriceps lesion | 2 out of 2 (T4 spine and kidney)  Pulmonary, quadriceps and RP lesion not assessed | Clear cell RCC, pT3a, nucleolar grade 2.  Bone biopsy – clear cell RCC. |

Abbreviations: IVC = Inferior vena cava, p=pathological, N=number, T = thoracic
